# Emergence of a Potent Multidrug Efflux Pump Variant That Enhances *Campylobacter* Resistance to Multiple Antibiotics

**DOI:** 10.1128/mBio.01543-16

**Published:** 2016-09-20

**Authors:** Hong Yao, Zhangqi Shen, Yang Wang, Fengru Deng, Dejun Liu, Gaowa Naren, Lei Dai, Chih-Chia Su, Bing Wang, Shaolin Wang, Congming Wu, Edward W. Yu, Qijing Zhang, Jianzhong Shen

**Affiliations:** a Beijing Advanced Innovation Center for Food Nutrition and Human Health, College of Veterinary Medicine, China Agricultural University, Beijing, China; b College of Veterinary Medicine, Iowa State University, Ames, Iowa, USA; c Department of Physics and Astronomy, Iowa State University, Ames, Iowa, USA; d Food Safety and Technology Department, University of Nebraska, Lincoln, Nebraska, USA

## Abstract

Bacterial antibiotic efflux pumps are key players in antibiotic resistance. Although their role in conferring multidrug resistance is well documented, the emergence of “super” efflux pump variants that enhance bacterial resistance to multiple drugs has not been reported. Here, we describe the emergence of a resistance-enhancing variant (named RE-CmeABC) of the predominant efflux pump CmeABC in *Campylobacter*, a major zoonotic pathogen whose resistance to antibiotics is considered a serious antibiotic resistance threat in the United States. Compared to the previously characterized CmeABC transporters, RE-CmeABC is much more potent in conferring *Campylobacter* resistance to antibiotics, which was shown by increased MICs and reduced intracellular accumulation of antibiotics. Structural modeling suggests that sequence variations in the drug-binding pocket of CmeB possibly contribute to the enhanced efflux function. Additionally, RE-CmeABC expands the mutant selection window of ciprofloxacin, enhances the emergence of antibiotic-resistant mutants, and confers exceedingly high-level resistance to fluoroquinolones, an important class of antibiotics for clinical therapy of campylobacteriosis. Furthermore, RE-CmeABC is horizontally transferable, shifts antibiotic MIC distribution among clinical isolates, and is increasingly prevalent in *Campylobacter jejuni* isolates, suggesting that it confers a fitness advantage under antimicrobial selection. These findings reveal a new mechanism for enhanced multidrug resistance and an effective strategy utilized by bacteria for adaptation to selection from multiple antibiotics.

## IMPORTANCE

Bacterial antibiotic efflux pumps are ubiquitously present in bacterial organisms and protect bacteria from the antibacterial effects of antimicrobials and other toxic compounds by extruding them out of cells. Thus, these efflux transporters represent an important mechanism for antibiotic resistance. In this study, we discovered the emergence and increasing prevalence of a unique efflux pump variant that is much more powerful in the efflux of antibiotics and confers multidrug resistance in *Campylobacter*, which is a major foodborne pathogen transmitted to humans via the food chain. Unlike other specific resistance determinants that only allow bacteria to resist a particular antimicrobial, the acquisition of a functionally enhanced efflux pump will empower bacteria with simultaneous resistance to multiple classes of antibiotics. These findings reveal a previously undescribed mechanism for enhanced multidrug resistance and open a new direction for us to understand how bacteria adapt to antibiotic treatment.

## INTRODUCTION

As evidenced by the recent emergence and spread of bacteria resistant to the last-resort antibiotics (e.g., carbapenems and colistin), the increasing prevalence of antibiotic-resistant pathogens has become a public health crisis (1–4). Different from other antibiotic resistance mechanisms, bacterial multidrug efflux pumps confer resistance to structurally diverse antimicrobials. Among various types of efflux transporters, members of the resistance-nodulation-cell division (RND) family are the most important in mediating antibiotic resistance in Gram-negative bacteria (5, 6). Typically, an RND efflux transporter is an inner membrane protein. It interacts with a periplasmic fusion protein and an outer membrane channel protein to assemble as a tripartite efflux complex that spans the entire cell envelope and extrudes antimicrobials directly out of bacterial cells (6). These RND efflux transporters are commonly controlled by regulatory factors, and overexpression of these efflux transporters is typically required for mediating clinically relevant levels of antibiotic resistance (5–10). Recently, Blair et al. reported that a single amino acid substitution in the drug-binding pocket of the AcrB transporter enhanced the function of the efflux pump and conferred clinically relevant resistance in *Salmonella* (11), suggesting an overexpression-independent mechanism for enhancing the function of a multidrug-resistant transporter.

Thermophilic *Campylobacter* species, including *Campylobacter jejuni* and *Campylobacter coli*, are major foodborne pathogens and are among the most frequent causes of bacterial gastroenteritis in humans, accounting for 400 million to 500 million cases of diarrhea each year worldwide (12). Transmission of *Campylobacter* bacteria to humans occurs mainly via the foodborne route, and contaminated poultry meat, water, and milk are the main sources of infection (13, 14). Because of the use of antibiotics in animal agriculture and medical settings, antibiotic-resistant *Campylobacter* is increasingly prevalent worldwide (15, 16). Because of its rising prevalence and impact on public health, antibiotic-resistant *Campylobacter* has been listed by the Centers for Disease Control and Prevention as a serious antibiotic resistance threat in the United States (17). In *Campylobacter* bacteria, the tripartite multidrug efflux pump CmeABC is the predominant efflux system and plays a key role in mediating resistance to structurally diverse antimicrobials (18). CmeABC constitutes the CmeB inner membrane transporter, belonging to the RND protein family; the CmeA periplasmic protein, a member of the membrane fusion protein family; and the CmeC outer membrane channel protein. This powerful efflux system is regulated primarily by CmeR and secondarily by CosR, both of which bind to the promoter region of the *cmeABC* operon and function as transcriptional repressors for this efflux system (19, 20). Notably, CmeABC is important for both intrinsic and acquired resistance to antibiotics, but overexpression of *cmeABC* confers only a modest increase in the MICs of antibiotics compared with the base-level expression of the efflux pump (19, 21).

In this study, we report the identification of a potent variant of CmeABC that shows an enhanced efflux function. We show that this variant CmeABC is highly potent against multiple antibiotics, promotes the emergence of antibiotic-resistant mutants, and confers exceedingly high-level resistance to fluoroquinolones. Additionally, we discovered that the *cmeABC* variant is increasingly prevalent in *C. jejuni* isolates, indicating that it confers on *Campylobacter* a fitness advantage under antibiotic selection pressure. To the best of our knowledge, this is the first report on the emergence of a “super” efflux pump variant that empowers bacteria with simultaneous resistance to multiple classes of drugs.

## RESULTS

### Identification of a unique CmeABC variant associated with resistance to florfenicol and other antimicrobials.

Florfenicol, the fluorinated derivative of chloramphenicol, has been used for treatment of respiratory diseases in food-producing animals, and previously we reported a high prevalence of florfenicol resistance in *Campylobacter* isolates in China (22). A *C. coli* isolate, DH161 (Table 1), was florfenicol resistant but did not harbor any known mechanisms (e.g., mutations in rRNA or acquisition of *floR*, *fexA*, *fexB*, *cfr*, or *optrA*) that confer florfenicol resistance, as determined by PCR (see Table S3 in the supplemental material). A natural-transformation assay revealed that florfenicol resistance in DH161 was transferrable between *C. coli* and *C. jejuni*. Specifically, the MIC of florfenicol for transformant NT161 was 32-fold higher than that for the recipient *C. jejuni* NCTC 11168 (Table 1). Notably, NT161 also showed increased resistance to other antibiotics, including chloramphenicol (16-fold), ciprofloxacin (8-fold), erythromycin (4-fold), and tetracycline (4-fold). These findings indicated that the transformation resulted in the transfer of multidrug resistance, suggesting that either multiple antibiotic resistance genes/mutations or a multidrug resistance mechanism was transferred from DH161 to NT161. Subsequently, random transposon mutagenesis of NT161 was conducted with EZ-Tn*5* to identify the specific mechanism responsible for florfenicol resistance in the transformant, which led to the identification of the transposon mutant NT161::*aphA-3*, which was susceptible to florfenicol. Determination of the transposon insertion site in NT161::*aphA-3* revealed the insertion of the EZ-Tn*5* transposon into a gene whose nucleotide sequence was 78.7% identical to that of the native *cmeB* gene in NCTC 11168. Notably, compared with NT161, NT161-*aphA-3* showed a marked decrease in the MICs of florfenicol (64-fold), chloramphenicol (64-fold), ciprofloxacin (>32-fold), erythromycin (>32-fold), and tetracycline (>64-fold) (Table 1). These results suggest that a *cmeABC*-related mechanism was likely to be responsible for the elevated resistance to florfenicol and other antimicrobials.

**TABLE 1:** Antimicrobial susceptibilities of the key *Campylobacter* strains used in this study

Strain	Description	MIC (mg/liter)^a^ of:				
		FFC	CHL	CIP	ERY	TET
DH161	*C. coli* from duck, isolated in 2009	16	256	256	4	512
NCTC 11168	*C. jejuni* reference strain	0.5	1	0.125	1	1
NT161	Transformant of NCTC 11168 carrying RE-*cmeABC* from DH161	16	16	1	4	4
NT161-*aphA-3*	NT161*cmeABC*::*aphA-3*	0.25	0.25	<0.03	<0.125	<0.06
11168-V1	NCTC 11168 with RE-*cmeABC* of NT161 and its promoter sequence	16	16	1	4	4
11168-V2	NCTC 11168 with RE-*cmeABC* of NT161 but original promoter of *cmeABC* in NCTC 11168	4	4	0.5	2	2
81–176	*C. jejuni* reference strain	0.5	2	0.125	0.25	-
81–176-V	81-176 with RE-*cmeABC* of DH161	32	>64	1	512	-
11168-C257T	NCTC 11168 with C257T mutation in *gyrA*	0.5	1	16	1	1
NT161-C257T	NT161 with C257T mutation in *gyrA*	16	16	256	4	4

a CHL, chloramphenicol; CIP, ciprofloxacin; ERY, erythromycin; FFC, florfenicol; TET, tetracycline.

To further examine this possibility, we performed whole-genome sequence analysis of NT161. Comparative analysis of the draft genome of NT161 with the complete genome of NCTC 11168 (GenBank accession number NC_002163) revealed that a 5,691-bp segment containing the entire *cmeABC* operon of DH161 replaced the same locus of NCTC 11168. Beyond this replacement, no other insertions/deletions or plausible single-nucleotide polymorphisms were observed in NT161. These results indicated that the natural transformation led to allelic exchange of *cmeABC* between the donor and recipient strains and acquisition of *cmeABC* from DH161 elevated multidrug resistance in NT161. Here we designate this CmeABC variant of DH161 RE-CmeABC (resistance-enhancing CmeABC).

### Functional confirmation of RE-CmeABC.

To verify the function of RE-CmeABC, we replaced the *cmeABC* operon in NCTC 11168 with RE-*cmeABC* by gene-specific replacement by natural transformation. PCR and Sanger sequencing confirmed the successful allelic replacement in the transformant. As shown in Table 1, transformant 11168-V1 showed 32-, 16-, 8-, 4-, and 4-fold increases in the MICs of florfenicol, chloramphenicol, ciprofloxacin, erythromycin, and tetracycline, respectively, compared to the recipient strain NCTC 11168 (Table 1). In addition, we introduced RE-*cmeABC* into a different *C. jejuni* strain, 81-176. We first deleted the whole *cmeABC* operon natively present in 81-176 and then introduced RE-*cmeABC* into a different locus via natural transformation. The 81-176 transformant with RE-*cmeABC* (named 81-176-V) showed a marked increase in the MICs of florfenicol (64-fold), chloramphenicol (>32-fold), and ciprofloxacin (8-fold) compared with 81-176 with its native *cmeABC* operon (Table 1). These results clearly show that RE-*cmeABC* confers significantly higher MICs of different antibiotics.

Sequence analysis of the promoter region of RE-*cmeABC* indicated the presence of an A → G mutation compared to the *cmeABC* promoter in NCTC 11168, resulting in an imperfect inverted repeat in the CmeR-binding site. Previous studies have indicated that the mutations in the CmeR-binding site result in overexpression of CmeABC in *Campylobacter* (20, 23). We compared the *cmeABC* expression levels in 11168-V1 and NCTC 11168 by real-time PCR. The results show a 5-fold increase in the expression of *cmeABC* in 11168-V1 over that in NCTC 11168. To differentiate the functional differences attributable to overexpression and sequence variation itself, we further constructed transformant 11168-V2 in which the original *cmeABC* coding region of NCTC 11168 was replaced with RE-*cmeABC* but it retained the native promoter sequence of NCTC 11168. Real-time quantitative reverse transcription (RT)-PCR showed that RE-*cmeABC* in 11168-V2 was expressed at a level similar (*P* > 0.05) to that of *cmeABC* in NCTC 11168. However, the MICs of florfenicol, chloramphenicol, ciprofloxacin, erythromycin, and tetracycline were 8-, 4-, 4-, 2-, and 2-fold higher, respectively, in 11168-V2 than in NCTC 11168 (Table 1). These MIC differences were attributable to sequence variations between the two *cmeABC* copies. Comparison of 11168-V1 with 11168-V2 revealed 4-fold (for phenicols) and 2-fold differences (for ciprofloxacin, erythromycin, and tetracycline), which were attributable to the increased expression of RE-*cmeABC*. Thus, both sequence variation and enhanced expression contributed to the resistance-enhancing function of RE-CmeABC.

**FIG 1:**
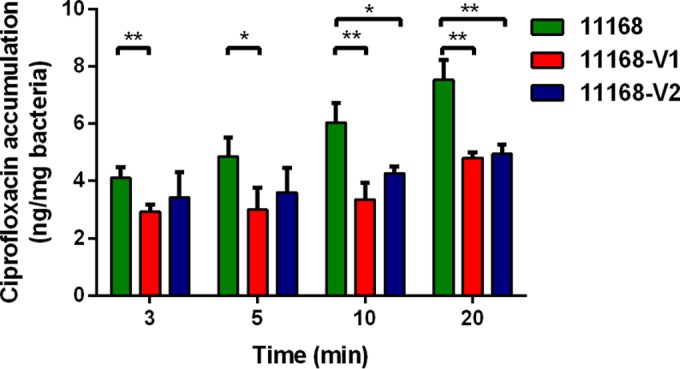
Accumulation of ciprofloxacin in NCTC 11168, 11168-V1, and 11168-V2. Each bar represents the mean and standard deviation of triplicate samples. A *t* test was used to perform the statistical analysis. *, *P* < 0.05; **, *P* < 0.01.

We further performed accumulation assays to assess whether RE-CmeABC extrudes more ciprofloxacin. Compared to NCTC 11168, 11168-V1 and 11168-V2 accumulated significantly less ciprofloxacin (Fig. 1), while there was no significant difference in ciprofloxacin accumulation between 11168-V1 and 11168-V2. This result indicated that RE-CmeABC indeed has an enhanced efflux function.

### Contribution of RE-CmeABC to extremely high-level ciprofloxacin resistance in *Campylobacter* isolates.

Fluoroquinolone-resistant *Campylobacter* is increasingly prevalent, and its resistance is mediated by GyrA mutations in conjunction with the function of CmeABC (15). Typically, fluoroquinolone-resistant *Campylobacter* isolates have ciprofloxacin MICs ranging from 4 to 32 mg/liter (24). However, we noticed that DH161 had an extremely high ciprofloxacin MIC (256 mg/liter; Table 1). In addition, multiple *C. jejuni* isolates in our previous surveillance work showed ciprofloxacin MICs of ≥256 mg/liter (see Table S1 in the supplemental material). These isolates harbored the known C257T (Thr-86-Ile) mutation in *gyrA*, but there were no plasmid-mediated quinolone resistance (PMQR) genes, as determined by PCR (see Table S3). Thus, the C257T mutation alone could not explain the extremely high ciprofloxacin MIC. We then sequenced and analyzed the CmeABC operon in these *C. jejuni* isolates. CmeA and CmeC in these isolates were homologous (>98% amino acid identities) to CmeA and CmeC in NCTC 11168, while CmeB in these *C. jejuni* strains showed only ~81% amino acid sequence identity to CmeB in NCTC 11168 but was highly homologous (>99.5% amino acid sequence identity) to CmeB in *C. coli* DH161, indicating that these *C. jejuni* isolates harbored RE-CmeABC. Given that RE-CmeABC showed an enhanced function in conferring antibiotic resistance, we hypothesized that the extremely high ciprofloxacin MIC was due to the synergistic action of the CmeABC variant and the *gyrA* mutation. To prove this possibility, the C257T mutation in *gyrA* was introduced into both NCTC 11168 and NT161, which differed only in the *cmeABC* operon. As shown in Table 1, the ciprofloxacin MIC for NT161 containing the C257T mutation was 256 mg/liter, while the ciprofloxacin MIC for NCTC 11168 containing the C257T mutation was 16 mg/liter, a 16-fold difference. These results strongly indicate that RE-CmeABC together with the C257T mutation conferred extremely high-level ciprofloxacin resistance.

### RE-CmeABC increases the frequencies of emergence of fluoroquinolone-resistant *Campylobacter* mutants.

Previous studies demonstrated that CmeABC contributes to the emergence of fluoroquinolone-resistant *Campylobacter* mutants (21, 25). Since RE-CmeABC, identified in this study, shows an enhanced efflux function compared with the typical CmeABC efflux pump, we speculated that RE-CmeABC could even further promote the emergence of fluoroquinolone-resistant *Campylobacter* mutants under selection pressure. As shown in Table S2 in the supplemental material, when ciprofloxacin was used at 1.25 or 4 mg/liter to select the mutants, the frequency of emergence of fluoroquinolone-resistant mutants was about 9-fold (*P* < 0.05) and 25-fold higher (*P* < 0.05), respectively, in NT161 than in NCTC 11168. Importantly, when a ciprofloxacin concentration of 8 or 16 mg/liter was used on the selective agar plates, no mutant colonies of NCTC 11168 were detected, while the frequency of mutant emergence in NT161 remained at ~10^−8^. These results clearly indicate that the *cmeABC* variant expanded the mutant selection window for fluoroquinolones and increased the frequency of emergence of fluoroquinolone-resistant mutants under antibiotic selection.

### Structural modeling of CmeB suggests enhanced drug binding by the transporter.

**FIG 2:**
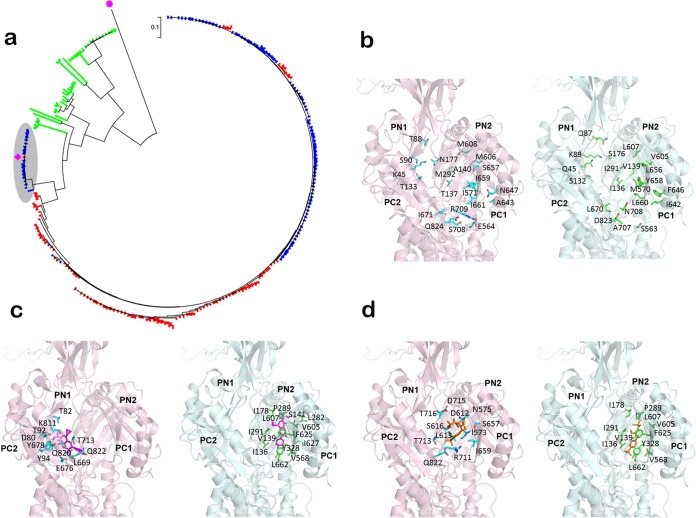
Sequence analysis and structure prediction of CmeB. (a) Phylogenetic analysis of *Campylobacter* CmeB sequences identified in this study and deposited in the GenBank database. Different *Campylobacter* species are shown in different colors as follows: red, *C. coli*; blue, *C. jejuni*; green, other *Campylobacter* species. The purple square indicates CmeB of isolate DH161. The unique subtree of RE-CmeB and its homologs from *C. jejuni* and *C. coli* is shaded gray. The purple dot indicates multidrug efflux RND transporter AcrB (accession number WP_044694729.1) from *E. coli*, which was used as the outgroup. (b) The CmeB structures predicted by the Modeller program. For clarity, only the predicted periplasmic domain structures of NCTC 11168 CmeB (pink) and RE-CmeB (DH161, light green) are shown. The 22 mutated residues located in the periplasmic drug-binding cavity of the mutant transporter are represented by green sticks. The corresponding residues in wild-type CmeB are represented by cyan sticks. (c) The predicted bound ciprofloxacin molecules are magenta. The residues involved in binding are represented by sticks. (d) The predicted bound florfenicol molecules are orange. The residues involved in binding are represented by sticks.

Generally, the CmeB amino acid sequences of *C. jejuni* and *C. coli* isolates are at least 93% identical (Fig. 2a), but CmeB in RE-CmeABC is divergent, showing ~81% homology with that of NCTC 11168. Interestingly, there are 18 CmeB homologs (from 18 different *C. jejuni* isolates) that are similar to CmeB in RE-CmeABC in the GenBank database, most of which were deposited in 2015. On the basis of phylogenetic analysis, CmeB in RE-CmeABC and its 18 homologs formed a unique subtree (shaded gray in Fig. 2a), which is apart from the majority of the CmeB sequences of *C. jejuni* and *C. coli* in the database.

To help understand how RE-CmeABC has an enhanced efflux function, we compared the predicted structures of CmeB in RE-CmeABC and CmeB in NCTC 11168 by homology modeling, based on the crystal structure of the MexB membrane protein (Protein Data Bank ID 2V50) (26) and with the Modeller program (27). Protein sequence alignment suggests that MexB is >40% identical to these two CmeB proteins. As expected, each predicted CmeB structure constitutes 12 transmembrane helices (TMs 1 to 12) and two large extracellular loops protruding out of the inner membrane and into the periplasmic space (Fig. 2b). These two periplasmic loops are located between TMs 1 and 2 and between TMs 7 and 8, respectively, generating a periplasmic domain of the pump. As in AcrB (28, 29) and MexB (26), this periplasmic domain can be divided into six subdomains (PN1, PN2, PC1, PC2, DN, and DC) (Fig. 2b). A cleft is formed between subdomains PC1 and PC2. This cleft is likely to create an entrance for different substrates, allowing them to enter the pump through the periplasm and outer leaflet of the inner membrane. Deep inside the cleft, the pump forms a large internal cavity. In AcrB, this cavity has been shown to form an important binding site that plays a predominant role in recognizing substrates for export. Interestingly, of the 198 amino acid differences between the NCTC 11168 and variant CmeB transporters, 22 are localized in this drug-binding cavity (Fig. 2b).

To understand if these mutated residues in the variant transporter are important for recognizing drug molecules, we used AutoDock Vina (30) to determine how CmeB binds drugs. Specifically, Vina was employed to predict the binding modes for ciprofloxacin and florfenicol. The data suggest that these two drugs are bound within the cavity between subdomains PC1 and PC2 in both CmeB and the variant transporter. However, these two transporters utilize different sets of residues to contact these drugs. In CmeB of NCTC 11168, residues D80, T82, T92, Y94, K811, L669, E676, Y678, T713, Q820, and Q822 are predicted to be involved in the binding of ciprofloxacin (Fig. 2c), while the variant transporter may use I136, V139, S141, I178, P289, I291, L282, V605, L607, F625, I627, Y328, V568, and L662 to bind ciprofloxacin (Fig. 2c). For florfenicol binding, CmeB of NCTC 11168 is likely to employ residues I573, N575, D612, L613, S616, S657, I659, T713, D715, R711, T716, and Q822 to interact with the drug (Fig. 2d); however, the variant transporter may utilize residues I178, I136, V139, P289, I291, Y328, V568, V605, L607, F625, and L662 to bind florfenicol (Fig. 2d). Interestingly, on the basis of the prediction, it was found that the variant transporter tends to use the mutated residues, including I136, V139, I291, V605, and L607, to bind these drugs. The ciprofloxacin- and florfenicol-binding energies of CmeB of NCTC 11168 are predicted to be −7.4 and −6.6 kcal/mol, respectively, while the variant transporter seems to secure these drugs more tightly, with predicted binding energies of −8.1 kcal/mol for ciprofloxacin and −7.1 kcal/mol for florfenicol. These findings suggest that the amino acid changes enhance drug binding in the *cmeB* variant.

### Rising prevalence of RE-CmeABC in *C. jejuni* isolates.

To understand the epidemiological significance of RE-CmeABC, we examined its distribution among 2,002 *Campylobacter* isolates (1,458 of *C. coli* and 544 of *C. jejuni*) derived from chickens and swine in China from 2012 to 2014 (Table 2). Among the isolates examined, 236 (11.8%) carried RE-CmeABC, including 189 *C. jejuni* and 47 *C. coli* isolates (Table 2). The percentage of *Campylobacter* isolates harboring RE-*cmeABC* increased from 7.0% (55/784) in 2012 to 9.8% (60/611) in 2013 and to 19.9% (121/607) in 2014. A chi-square test revealed a significant linear trend in the ordered years 2012 to 2014 (*P* < 0.0001), indicating an emergent trend of the RE-CmeABC variant in *Campylobacter* isolates in China. Remarkably, RE-*cmeABC* was highly prevalent in the *C. jejuni* isolates: 30.8% (37/120) in 2012, 16.7% (44/264) in 2013, 67.5% (108/160) in 2014, and 34.7% (189/544) overall (Table 2). The chi-square test also revealed a significant linear trend in the ordered years 2012 to 2014 (*P* < 0.0001). The decreased incidence in 2013 might be due to the effect of sampling bias, as more than half (146) of the *C. jejuni* isolates were from Ningxia Province, where the prevalence of RE-*cmeABC* was much lower than that in the isolates from other regions (Table 2). These results indicated that RE-*cmeABC* is increasingly prevalent in *C. jejuni* isolates in China. On the contrary, the percentage of *C. coli* isolates containing RE-*cmeABC* was low (ranging from 2.7 to 4.6%) and did not show a significant linear trend during 2012 to 2014 (*P* = 0.73).

**TABLE 2:** Distribution of RE-*cmeABC* in various *Campylobacter* isolates

Yr and location of isolation	Host	(%) no. of positive isolates/total no. of isolates	(%) no. of positive isolates/total no. of isolates	
			*C. jejuni*	*C. coli*
2012				
Guangdong	Chicken	(8.9) 11/124	(20.6) 7/34	(4.4) 4/90
	Swine	(1.0) 2/191	(0.0) 0/1	(1.1) 2/190
Ningxia	Chicken	(15.2) 5/33	(31.3) 5/16	(0.0) 0/17
	Swine	(1.4) 3/208		(1.4) 3/208
Shandong	Swine	(2.8) 2/71		(2.8) 2/71
Shanghai	Chicken	(20.4) 32/157	(36.2) 25/69	(8.0) 7/88
Total		(7.0) 55/784	(30.8) 37/120	(2.7) 18/664
2013				
Guangdong	Chicken	(17.0) 18/106	(66.7) 10/15	(8.8) 8/91
	Swine	(3.7) 2/54	(100.0) 1/1	(1.9) 1/53
Ningxia	Chicken	(0.5) 1/196	(0.7) 1/144	(0.0) 0/52
	Swine	(0.0) 0/24	(0.0) 0/2	(0.0) 0/22
Shandong	Chicken	(25.0) 18/72	(19.4) 13/67	(100.0) 5/5
Shanghai	Chicken	(9.8) 13/132	(40.7) 11/27	(1.9) 2/105
Henan	Chicken	(80.0) 8/10	(100.0) 8/8	(0.0) 0/2
	Swine	(0.0) 0/17		(0.0) 0/17
Total		(9.8) 60/611	(16.7) 44/264	(4.6) 16/347
2014				
Guangdong	Chicken	(15.6) 21/135	(81.3) 13/16	(6.7) 8/119
Ningxia	Chicken	(6.7) 4/60	(8.1) 3/37	(4.3) 1/23
	Swine	(30.8) 8/26	(66.7) 4/6	(20.0) 4/20
Shandong	Chicken	(0.0)0/164	(0.0) 0/1	(0.0) 0/163
Shanghai	Chicken	(18.2) 14/77	(56.0) 14/25	(0.0) 0/52
	Swine	(0.0) 0/52		(0.0) 0/52
Henan	Chicken	(93.7) 74/79	(98.7) 74/75	(0.0) 0/4
	Swine	(0.0) 0/14		(0.0) 0/14
Total		(19.9) 121/607	(67.5) 108/160	(2.9) 13/447
All				
	Chicken	(16.3) 219/1,345	(34.4) 184/534	(4.3) 35/811
	Swine	(2.6) 17/657	(50.0) 5/10	(1.9) 12/647
Total		(11.8) 236/2,002	(34.7) 189/544	(3.2) 47/1,458

**FIG 3:**
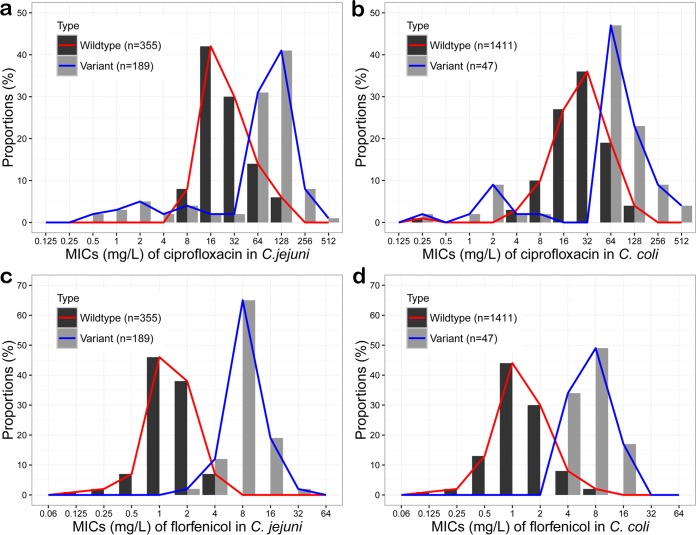
Distribution of ciprofloxacin and florfenicol MICs for *Campylobacter* isolates with RE-*cmeABC* or other CmeABC types. (a) Distribution of ciprofloxacin MICs for *C. jejuni*. (b) Distribution of ciprofloxacin MICs for *C. coli*. (c) Distribution of florfenicol MICs for *C. jejuni*. (d) Distribution of florfenicol MICs for *C. coli*. In all panels, “Variant” indicates strains that contain RE-*cmeABC* while “Wildtype” depicts isolates containing a *cmeABC* operon that is not RE-*cmeAB*C.

We further analyzed the influence of RE-CmeABC on the distribution of MICs of ciprofloxacin and florfenicol among the 2,002 *Campylobacter* isolates (16). Notably, the isolates containing RE-*cmeABC* or other types of *cmeABC* showed a bimodal pattern of distribution (Fig. 3). This is especially obvious with *C. jejuni* and the antibiotic florfenicol. The isolates harboring RE-CmeABC shifted the MIC distribution to the higher range. A Fisher exact test confirmed that the MICs of ciprofloxacin and florfenicol were significantly higher for the isolates containing RE-CmeABC than for other isolates (*P* < 0.0001). These results indicate that RE-CmeABC increases antibiotic MICs at the population level.

### Molecular typing and phylogenetic analysis of RE-*cmeABC*-carrying *C. jejuni* and *C. coli* isolates.

**FIG 4:**
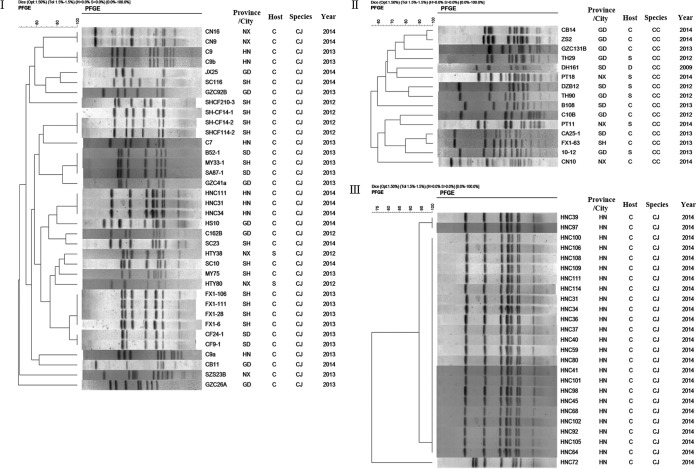
PFGE analysis of representative RE-*cmeABC*-positive *Campylobacter* isolates. SmaI was used for digestion. The regions of isolation (provinces and cities) include Guangdong (GD), Shandong (SD), Ningxia (NX), Henan (HN), and Shanghai (SH). The host species include chickens (C), swine (S), and ducks (D). (I) Representative RE-*cmeABC*-positive *C. jejuni* (CJ) isolates (*n* = 36). With 80% genetic similarity as a cutoff, the *C. jejuni* isolates were grouped into 17 clusters (PFGE patterns represented by multiple strains) and 24 unique PFGE patterns. (II) Representative RE-*cmeABC*-positive *C. coli* (CC) isolates (*n* = 15). *C. coli* isolates were grouped into 11 clusters and 15 unique PFGE patterns. (III) Representative RE-*cmeABC*-positive *C. jejuni* isolates from Henan Province in 2014 (*n* = 25). The isolates were grouped into two clusters and three unique PFGE patterns.

To determine if RE-*cmeABC*-carrying *Campylobacter* isolates were genetically related, 51 isolates (36 of *C. jejuni* and 15 of *C. coli*) representing different regions, years, host species, and antimicrobial susceptibility patterns were selected for pulsed-field gel electrophoresis (PFGE) analysis by SmaI digestion (Fig. 4I and II). With 80% genetic similarity as the cutoff, the 36 *C. jejuni* isolates were grouped into 17 clusters (PFGE patterns represented by multiple strains) and 24 unique PFGE patterns (PFGE patterns represented by a single strain) (Fig. 4I), while the 15 *C. coli* isolates were grouped into 11 clusters and 15 unique PFGE patterns (Fig. 4II). In general, the *C. jejuni* isolates from different regions or different years were genetically diverse; however, identical PFGE patterns were obtained with some isolates derived from the same region and the same year. Some examples included the four isolates from Shanghai (Fx1-106, Fx1-111, Fx1-6, and Fx-1-28) and the three isolates from Henan (HNC111, HNC31, and HNC34) (Fig. 4I). Additionally, we did a more detailed analysis of the RE-*cmeABC*-positive *C. jejuni* isolates collected in 2014 from Henan and found that most of them had the same PFGE pattern (Fig. 4III). These results indicated that RE-*cmeABC*-carrying isolates showed both overall genetic diversity and regional clonality, suggesting that both clonal expansion and horizontal transmission were involved in the dissemination of this *cmeABC* variant.

## DISCUSSION

In this study, we identified an emergent CmeABC variant (RE-CmeABC) that is more potent in the efflux of antibiotics, conferring enhanced resistance to multiple antimicrobials, especially to florfenicol and fluoroquinolones (Fig. 1; Table 1). Additionally, RE-CmeABC in conjunction with the C257T mutation in *gyrA* confers exceedingly high-level resistance to ciprofloxacin (MIC, ≥256 mg/liter). This RE-*cmeABC* variant is especially prevalent in *C. jejuni* and is emergent in chickens and swine in China, suggesting that it facilitates the adaptation of *Campylobacter* in the food-producing environment, where antibiotics are frequently used. Genotyping analysis suggested that both clonal expansion and horizontal transmission are involved in the spread of this unique CmeABC variant.

The elevated resistance to multiple antimicrobials seen is due to both overexpression and sequence variation of RE-*cmeABC*. Previous studies indicated that overexpression of *cmeABC* conferred only a moderate, usually 2-fold, increase in the MICs of several antimicrobials (19, 31). In this study, however, overexpressed RE-*cmeABC* resulted in 32- and 8-fold increases in the MICs of florfenicol and ciprofloxacin, respectively (Table 1), indicating a much enhanced function of RE-CmeABC. Similar to well-characterized AcrB (32), CmeB is responsible for the substrate recognition and interaction in the CmeABC efflux system. Thus, the sequence variations in CmeB likely had the most impact on its function. Indeed, structural modeling predicted that ciprofloxacin and florfenicol are bound within the cavity between subdomains PC1 and PC2 in both CmeB and its variant transporter RE-CmeABC (Fig. 2). However, these two transporters utilize different sets of residues to contact these drugs. On the basis of the prediction, it was found that the variant transporter tends to use the mutated residues, including I136, V139, I291, V605, and L607, to bind these drugs, which secure these drugs more tightly than the typical CmeB protein. Thus, the enhanced function of CmeB in RE-CmeABC may be explained, at least in part, by its altered antibiotic-binding kinetics. This possibility remains to be verified by structural analysis in future studies.

Results of this study also reveal a clear rising trend in the prevalence of RE-CmeABC in *C. jejuni* (Table 2). The difference in the prevalence of RE-*cmeABC* between *C. jejuni* and *C. coli* is probably due to the fact that *C. coli* is intrinsically more resistant to antimicrobials than *C. jejuni* is (16). Thus, under antimicrobial selection, *C. jejuni* may have evolved to acquire RE-*cmeABC* as a means of enhanced resistance and adaptation. Previously, Cagliero et al. reported that a multidrug-resistant *C. jejuni* strain (154KU) isolated in France harbored a *cmeB* gene that was quite divergent from the *cmeB* genes of other *Campylobacter* strains. Interestingly, the CmeB amino acid sequence of *C. jejuni* 154KU was 99.3% identical to that of RE-CmeABC identified in this study, indicating that it is a homolog of RE-CmeABC (33). This finding and the RE-CmeABC homologs recently deposited in GenBank from different countries suggest that this potent variant has emerged in other countries beyond China. The emergence and increasing prevalence of RE-CmeABC in China were likely driven by the extensive use of antimicrobials in animal production. Florfenicol, fluoroquinolones, macrolides, and tetracyclines are commonly used to prevent and control bacterial diseases in food animal production in China (16). Because of its enhanced function in multidrug resistance, harboring RE-CmeABC provides *Campylobacter* with an enhanced ability to adapt in the animal production environment.

Interestingly, PFGE analysis of the RE-CmeABC-harboring isolates indicated that they were genetically diverse, despite certain clonality among isolates from the same region and the same year. This finding suggests that RE-*cmeABC* can spread by horizontal gene transfer. Indeed, we showed under laboratory conditions that RE-*cmeABC* was easily transferred between *Campylobacter* isolates by natural transformation. Given that *Campylobacter* is naturally transformable, it is expected that RE-*cmeABC* will continue to increase in prevalence under selection pressure from antimicrobial use.

In conclusion, our findings reveal a “super” CmeABC variant utilized by *Campylobacter* for enhanced multidrug resistance. It desensitizes *Campylobacter* to multiple classes of antibiotics and promotes the emergence of fluoroquinolone-resistant mutants under selection pressure. Additionally, RE-CmeABC, together with GyrA mutations, confers exceedingly high-level resistance to fluoroquinolone, a key antibiotic used for the treatment of *Campylobacter* infections. Thus, dissemination of RE-CmeABC will likely have a significant impact on the clinical treatment of campylobacteriosis. Given that RE-*cmeABC* can be transferred by natural transformation, it is possible that this variant will continue to spread in clinical isolates. Thus, enhanced efforts are needed to prevent its further dissemination. Furthermore, our study reveals an effective strategy utilized by bacteria for adaptation to antibiotic selection. Gaining a specific resistance determinant only allows bacteria to resist a particular antimicrobial, while acquisition of a functionally enhanced efflux pump empowers bacteria with simultaneous resistance to multiple classes of antibiotics. Such a mechanism provides an efficient means for bacterial adaptation to selection pressure from the use of diverse antimicrobials.

## MATERIALS AND METHODS

### *Campylobacter* strains and antimicrobial susceptibility testing.

*C. coli* DH161 was isolated from a duck slaughterhouse in Shandong Province, China, in 2009. Additionally, a total of 2,002 *Campylobacter* isolates (1,458 of *C. coli* and 544 of *C. jejuni*) were tested for the presence of RE-*cmeABC* (Table 2). These *Campylobacter* strains were isolated from cecal contents, carcasses, and feces of swine and chickens from Shandong, Henan, Ningxia, Guangdong, and Shanghai under the surveillance program for antimicrobial resistance in *Campylobacter* of animal origin during 2012 to 2014 (16, 18). All of the *Campylobacter* strains were grown on Mueller-Hinton (MH) agar (Sigma-Aldrich, St. Louis, MO) at 42°C under microaerobic conditions (5% O_2_, 10% CO_2_, 85% N_2_).

Antimicrobial susceptibility testing was conducted by the standard agar dilution method according to the guidelines of the Clinical and Laboratory Standards Institute (34). *C. jejuni* ATCC 33560 was used for quality control. All of the experiments described above were repeated three times.

### Detection of florfenicol and fluoroquinolone resistance determinants.

Mutations involved in florfenicol resistance in the 23S rRNA gene and L4 and L22 ribosomal protein genes in the strains used in this study were detected by PCR and DNA sequencing. In addition, the *floR*, *fexA*, *fexB*, *cfr*, and *optrA* genes, known to confer resistance to florfenicol, were examined by PCR. The primers used are listed in Table S3 in the supplemental material. A mismatch amplification mutation assay (MAMA)-PCR was performed to detect the C257T (Thr-86-Ile) mutation in the quinolone resistance-determining region of the *gyrA* gene (35). Moreover, the strains were screened for the presence of *parC* and PMQR genes by PCR amplification with the primers in Table S3.

### Natural transformation.

Natural transformation was conducted as described previously (36). Briefly, genomic DNA of *C. coli* DH161 served as the donor DNA and *C. jejuni* NCTC 11168, susceptible to florfenicol, was used as the recipient strain. The transformants (including NT161) were selected on MH agar plates containing florfenicol (4 mg/liter). Transformation without donor DNA was used as a negative control. Transformants were examined for known mutations involved in florfenicol resistance.

The *cmeABC* operon in NCTC 11168 was replaced with RE-*cmeABC* of DH161 by natural transformation. The donor DNA was the PCR product of the entire RE-*cmeABC* operon. Briefly, RE-*cmeABC* was amplified from DH161 with primers CmeABC-F and CmeABC-R (see Table S3 in the supplemental material) and cloned into the pMD19-T vector (TaKaRa). This suicide vector was then transferred into *C. jejuni* NCTC 11168 by natural transformation (36), and the transformants (11168-V1 and 11168-V2) were selected on MH agar plates containing florfenicol (2 to 4 mg/liter). 11168-V1 and 11168-V2 have the same RE-*cmeABC* coding sequence, while 11168-V1 has the same promoter as DH161 and 11168-V2 has the same promoter as NCTC 11168. PCR and DNA sequencing were performed to confirm the presence of RE-*cmeABC* in these transformants. The three copies of 23S rRNA, as well as the L4 and L22 ribosomal protein genes, were also examined for potential mutations by PCR and DNA sequencing.

### Random transposon mutagenesis.

To identify the determinant involved in florfenicol resistance, we conducted random mutagenesis of transformant NT161 with the EZ-Tn*5* <KAN-2> Insertion kit (Epicentre Biotechnologies) (18). The transposon mutants were screened on MH agar plates containing kanamycin (50 mg/liter). Clones selected on the kanamycin plates were inoculated simultaneously onto two types of MH agar plates containing kanamycin (50 mg/liter) and florfenicol (4 mg/liter), respectively. Clones that failed to grow on plates containing 4 mg/liter florfenicol but grew on plates containing kanamycin at 50 mg/liter were selected to confirm the kanamycin resistance gene *aphA-3* insertion by PCR and its flanking regions by a modified random primer walking sequencing method (37).

### Whole-genome sequencing.

The whole genome of transformant NT161 was sequenced on an Illumina HiSeq 2000 platform at the Hudson Alpha Institute (Huntsville, AL). The backbone genome sequence of NT161 is NCTC 11168.

### Introduction of RE-*cmeABC* into *C. jejuni* 81-176.

The native *cmeABC* operon in *C. jejuni* 81-176 was deleted and replaced with a chloramphenicol resistance gene (*cat*) by the homologous-recombination method (19). The intact RE-*cmeABC* operon from DH161 was amplified with primers cmeAKpnI-F and cmeCKpnI-R. The amplicon was digested with KpnI and ligated into the corresponding sites of plasmid pUC-*cadF*-*ermB*-*cj1476c* (38). pUC-*cadF*-*ermB*-*cj1476c* contains a macrolide resistance gene (*ermB*) between the sequences homologous to the *cadF* and *cj1476c* genes. The constructed suicide plasmid pUC-c*adF-*RE-*cmeABC-ermB-cj1476c* was used to introduce RE-*cmeABC* into the 81-176 *cmeABC* deletion mutant strain. The transformants (81-176-V) were selected on MH agar plates containing erythromycin (10 mg/liter) and verified by PCR and DNA sequence analysis.

### Selection of the C257T mutation in the *gyrA* gene.

In order to assess the level of resistance to fluoroquinolones in strains coharboring RE-*cmeABC* and the DNA gyrase C257T mutation, the C257T mutation in *gyrA* was selected in *C. jejuni* NT161 and NCTC 11168 by exposure to ciprofloxacin. Briefly, strains NCTC 11168 and NT161 were grown overnight on antibiotic-free MH agar and then resuspended in MH broth to achieve a density of 10^9^ CFU/ml. Each suspension of 100 µl was separately spread onto MH agar plates supplemented with ciprofloxacin (4 mg/liter) and cultivated at 42°C for 2 to 3 days under microaerophilic conditions. Colonies on the plates were selected, and the C257T mutation in *gyrA* was confirmed by MAMA-PCR (35).

### Determining frequencies of emergence of fluoroquinolone-resistant mutants.

The frequency of emergence of fluoroquinolone-resistant *Campylobacter* mutants was detected as described previously (25). Briefly, strains NCTC 11168 and NT161 were grown on MH agar plates for 24 h at 42°C under microaerobic conditions (5% O_2_, 10% CO_2_, 85% N_2_). The cells were collected and suspended in 500 µl of MH broth. The total CFU count was determined by serial dilution and plating. The same amount (100 µl) of a bacterial suspension of each strain was plated onto MH agar plates containing four different concentrations of ciprofloxacin, i.e., 1.25, 4, 8, and 16 mg/liter, respectively. The frequency of emergence of fluoroquinolone-resistant mutants was calculated as the ratio of the CFU counts on ciprofloxacin-containing MH agar plates to the CFU counts on ciprofloxacin-free MH agar plates after 48 h of incubation at 42°C under microaerobic conditions. This experiment was repeated three times.

### Sequence analysis and structure prediction of CmeB.

The Protein Basic Local Alignment Search Tool (BLASTP) was used to search *Campylobacter* CmeB protein sequences (taxid:194 and taxid:72294) in the database of nonredundant protein sequences. The maximum number of target sequences was set at 250. Three sequences were eliminated from the analysis because of the low query coverage. A total of 247 sequences from *C. jejuni* (*n* = 105), *C. coli* (*n* = 98), and other species (*n* = 44) were used for further analysis. ClustalW was used to carry out multiple-sequence alignments of the 247 searched sequences together with CmeB of DH161 and a multidrug efflux RND transporter (accession no. WP_044694729.1) from *E. coli* (used as the outgroup). The maximum-likelihood phylogenetic tree was constructed with MEGA6.

The structures of CmeB in NCTC 11168 and DH161 were predicted by homology modeling with the Modeller program (27). The predictions were based on profile-profile sequence alignments (FFAS03) (39) by utilizing the structure of MexB (2V50) (26) as the template. The predicted structures were idealized with PHENIX (40) and then input into AutoDock Vina (version 1.1.2) (30). The structures of both ligands, ciprofloxacin and florfenicol, were obtained from the PubChem database (http://pubchem.ncbi.nlm.nih.gov). AutoDockTools (41) was then used to prepare the ligand files. The protein was set as a rigid structure, whereas the conformation of each ligand was optimized during all modeling and docking procedures. The iterated local search global optimizer algorithm was employed to predict the binding free energies for these compounds.

### Real-time RT-PCR analysis of *cmeABC* transcription.

Real-time RT-PCR was conducted to determine the expression levels of *cmeABC* in various strains as described previously (31). Briefly, NCTC 11168, 11168-V1, and 11168-V2 were grown in MH broth for 16 h at 42°C under microaerobic conditions. Two volumes of Sample Protector for RNA/DNA (TaKaRa) were added to the cultures to stabilize the total bacterial RNA. The mixtures were incubated at room temperature for 5 min, and then bacterial cells were collected by centrifugation at 5,000 × *g* for 10 min. Total RNA was purified by using an RNeasy minikit (Qiagen) according to the instructions included. Purified RNA was treated with TURBO DNA-free (Ambion) to remove DNA contamination. The primers used for real-time PCR are shown in Table S4 in the supplemental material. The two pairs of primers were designed to target the conserved regions of *cmeA* and *cmeC*, respectively, in NCTC 11168, 11168-V1, and 11168-V2. Ten-fold dilution series between 2.5 pg and 25 ng were made for RNA templates of NCTC 11168, 11168-V1, and 11168-V2 to generate the standard curve. RT-PCR assays were performed with the One Step SYBR PrimeScript PLUS RT-PCR kit (Perfect Real Time) (TaKaRa). The thermal cycling conditions were as follows: 10 min at 50°C, 5 min at 60°C, 5 min at 95°C, and then 40 cycles of 10 s at 95°C and 30 s at 58°C. Samples were normalized by using 16S RNA as an internal standard. Three biological replicates were prepared for each strain, and three technical replicates were performed for each RNA template. The relative changes (*n*-fold) in *cmeABC* transcription between *cmeABC* variant strains (11168-V1 and 11168-V2) and NCTC 11168 were calculated by the 2^−ΔΔ*CT*^ method as described previously (42).

### Ciprofloxacin accumulation assay.

The ciprofloxacin accumulation assay used was based on the method described previously (18). Overnight broth culture of *C. jejuni* was harvested and washed once in phosphate-buffered saline (PBS; pH 7.2), and the cell density was adjusted to an optical density at 600 nm of 20. Three different tubes were prepared for each sample. A cell suspension of each strain was incubated for 10 min at 37°C in a normal atmosphere, and then ciprofloxacin was added to a final concentration of 10 mg/liter. Aliquots (0.5 ml) were taken after 3, 5, 10, and 20 min and immediately diluted with 2.5 ml of ice-cold PBS. Bacterial cells were harvested by centrifugation at 4°C at 6,000 × *g* for 5 min and washed once with 2 ml of ice-cold PBS. Bacterial cells were resuspended in 0.2 ml of 0.1 M glycine hydrochloride (pH 3.0) and shaken at room temperature for 16 h. The supernatant was taken after centrifugation at 15,000 × *g* for 5 min, and fluorescence was measured with FLUOstar Omega with excitation and emission wavelengths of 279 and 447 nm, respectively. A standard curve was prepared by measuring the fluorescence emitted from 0.1 M glycine hydrochloride containing serially diluted ciprofloxacin and used to determine the concentration of ciprofloxacin in the samples.

### Detection of RE-*cmeABC* in *Campylobacter* isolates.

To measure the distribution of RE-*cmeABC* in *Campylobacter* isolates, primers BV-F (5′-CGTATTGCACGATTATTTGGAC) and BV-R (5′-ATCGTTATCAAACCCTCTATGTGCC) were designed according to the sequences specific for the *cmeB* variant in DH161 and used to specifically amplify the *cmeABC* variant. The PCR cycling conditions were as follows: initial denaturation at 95°C for 5 min; 30 cycles of 95°C for 30 s, 59°C for 30 s, and 72°C for 40 s; and a final extension at 72°C for 7 min. NCTC 11168 was used as a negative control, while DH161 was used as a positive control in the PCRs. Representative PCR products were selected for DNA sequencing analysis for verification.

### PFGE.

Representative *Campylobacter* isolates containing RE-*cmeABC* were genotyped by PFGE, which was conducted with a CHEF-DR III apparatus (Bio-Rad Laboratories, Hercules, CA) in accordance with the protocol for *Campylobacter* (43). The DNA of *Campylobacter* was digested with SmaI, and the DNA of *Salmonella* H9812 digested with XbaI was used as the reference marker. The results were analyzed with the InfoQuest FP software version 4.5 (Bio-Rad Laboratories).

### Accession number.

The *cmeABC* variant sequences of the strains involved in this study have been submitted to GenBank under accession number KT778507.

## SUPPLEMENTAL MATERIAL

Table S1  Antimicrobial susceptibilities of *Campylobacter* isolates that show exceedingly high-level resistance to fluoroquinolones. Table S1, DOCX file, 0.02 MB

Table S2  Frequencies of emergence of fluoroquinolone-resistant *Campylobacter* mutants under different ciprofloxacin selection pressures. Table S2, DOCX file, 0.01 MB

Table S3  Key primers used for PCRs in this study. Table S3, DOCX file, 0.04 MB

Table S4  Primers used for real-time RT-PCRs in this study. Table S4, DOCX file, 0.02 MB
