# Supplementary material for: Emergence of a Potent Multidrug Efflux Pump Variant That Enhances Campylobacter Resistance to Multiple Antibiotics
Source: mBio. 2016 Sep 20;7(5):e01543-16. doi: 10.1128/mBio.01543-16 (PMC5030363; doi:10.1128/mBio.01543-16)
Supplement: Table S1 — Antimicrobial susceptibilities of Campylobacter isolates that show exceedingly high-level resistance to fluoroquinolones. [file mbo005163000st1.docx]

**Table** **S1.** Antimicrobial susceptibility of *Campylobacter* isolates that show exceedingly high-level resistance to fluoroquinolones

| Strains *^a^* | Province/city *^b^* | Sample | Year of isolation | MIC (mg/L) *^c^* | | | | |
| --- | --- | --- | --- | --- | --- | --- | --- | --- |
|  |  |  |  | CIP | FFC | CHL | ERY | TET |
| DH161 | SD | Feces | 2009 | 256 | 16 | 16 | 4 | 512 |
| SH-CF14-1 | SH | Feces | 2012 | 512 | 16 | 128 | 4 | 512 |
| SH-CF114-2 | SH | Feces | 2012 | 512 | 16 | 64 | 4 | 512 |
| SH-CF14-2 | SH | Feces | 2012 | 512 | 16 | 64 | 4 | 512 |
| SH-CMC29 | SH | Retail broiler meats | 2012 | 256 | 16 | 16 | 256 | 512 |
| FX-1-28 | SH | Feces | 2013 | 256 | 16 | 16 | 4 | 64 |
| FX-1-111 | SH | Feces | 2013 | 256 | 16 | 16 | 4 | 64 |
| FX-1-106 | SH | Feces | 2013 | 256 | 8 | 16 | 16 | 32 |
| FX-1-89 | SH | Feces | 2013 | 256 | 8 | 16 | 16 | 32 |
| FX-1-6 | SH | Feces | 2013 | 256 | 8 | 16 | 4 | 64 |
| CF24-1 | SD | Feces | 2013 | 512 | 16 | 64 | 16 | 64 |
| CF9-1 | SD | Feces | 2013 | 512 | 16 | 64 | 16 | 64 |
| HNC100 | HN | Feces | 2014 | 256 | 8 | 16 | 4 | 256 |
| SC10 | SH | Feces | 2014 | 256 | 8 | 16 | 4 | 4 |
| SC86 | SH | Feces | 2014 | 256 | 8 | 16 | 4 | 4 |

*^a^* The strains except DH161 are all *C. jejuni* isolates of chicken origin. DH161 is a *C. coli* isolate from duck.

*^b^* SH, Shanghai; SD, Shandong; HN, Henan.

*^c^* CIP, ciprofloxacin; FFC, florfenicol; CHL, chloramphenicol; ERY, erythromycin; TET, tetracycline;
