# Supplementary material for: Emergence of a Potent Multidrug Efflux Pump Variant That Enhances Campylobacter Resistance to Multiple Antibiotics
Source: mBio. 2016 Sep 20;7(5):e01543-16. doi: 10.1128/mBio.01543-16 (PMC5030363; doi:10.1128/mBio.01543-16)
Supplement: Table S2 — Frequencies of emergence of fluoroquinolone-resistant Campylobacter mutants under different ciprofloxacin selection pressures. [file mbo005163000st2.docx]

**Table S2** Frequencies of emergence of fluoroquinolone-resistant *Campylobacter* mutants under different ciprofloxacin selection pressures

| Ciprofloxacin levels  (mg/L) | Frequency of mutant emergence*^a^* | |
| --- | --- | --- |
|  | NCTC 11168 | NT161 |
| 1.25 | 2.77×10^-8^±1.30×10^-8^ | 2.45×10^-7^±0.16×10^-8**^ |
| 4 | 2.88×10^-9^±1.60×10^-9^ | 7.19×10^-8^±2.43×10^-8*^ |
| 8 | 0 | 6.34×10^-8^±3.75×10^-8^ |
| 16 | 0 | 5.88×10^-8^±2.06×10^-8^ |

*^a^* Means ± standard deviations for three independent experiments. T-test was used to analyze the differences in frequencies of mutant emergence between NT161 and NCTC 11168. * *P*<0.05; ** *P*<0.01.
