# Supplementary material for: Emergence of a Potent Multidrug Efflux Pump Variant That Enhances Campylobacter Resistance to Multiple Antibiotics
Source: mBio. 2016 Sep 20;7(5):e01543-16. doi: 10.1128/mBio.01543-16 (PMC5030363; doi:10.1128/mBio.01543-16)
Supplement: Table S3 — Key primers used for PCRs in this study. [file mbo005163000st3.docx]

**Table** **S3** Key primers used for PCR in this study

| Primers | | Sequence (5’-3’) | References |
| --- | --- | --- | --- |
| 23S  rRNA | 23S-F | AGCTACTAAGAGCGAATGGT | ([1](#_ENREF_1)) |
|  | 23S-R | AAAGATAAGCCAAACGCTCT |  |
| L4 | L4-F | GTAGTTAAAGGTGCAGTACCA | ([2](#_ENREF_2)) |
|  | L4-R | GCGAAGTTTGAATAACTACG |  |
| L22 | L22-F | GAATTTGCTCCAACACGC | ([2](#_ENREF_2)) |
|  | L22-R | ACCATCTTGATTCCCAGTTTC |  |
| *floR* | floR-F | TCCTGAACACGACGCCCGCTAT | ([3](#_ENREF_3)) |
|  | floR-F | TCCTGAACACGACGCCCGCTAT |  |
| *fexA* | fexA-F | TTGGGAAGAATGGTTCAGGG | ([4](#_ENREF_4)) |
|  | fexA-R | ATCGGCTCAGTAGCATCACG |  |
| *fexB* | fexB-F | CCAACGCCAATACAACCA | This study |
|  | fexB-R | TCCTGTTCTCGGTGTGAT |  |
| *cfr* | cfr-F | TAAGAAGTAATAATGAGC | ([4](#_ENREF_4)) |
|  | cfr-R | TATAGAAGTCTACGAGG |  |
| *optrA* | A-F | AGGTGGTCAGCGAACTAA | ([5](#_ENREF_5)) |
|  | A-R | ATCAACTGTTCCCATTCA |  |
| *gyrA* | gyrA-cj-F | TTTTTAGCAAAGATTCTGAT | ([6](#_ENREF_6)) |
|  | gyrA-cj-R | CAAAGCATCATAAACTGCAA |  |
| *parC* | parC-F | TGGGATCCAAACCTGTTCAGCGCCGCATT | ([7](#_ENREF_7)) |
|  | parC-R | CGGAATTCGTGGTGCCGTTAAGCAAA |  |
| PMQR | qnrA-F | TTCAGCAAGAGGATTTCTCA | ([8](#_ENREF_8)) |
|  | qnrA-R | GGCAGCACTATTACTCCCAA |  |
|  | qnrB-F | CCTGAGCGGCACTGAATTTAT | ([8](#_ENREF_8)) |
|  | qnrB-R | GTTTGCTGCTCGCCAGTCGA |  |
|  | qnrC-F | GGGTTGTACATTTATTGAATC | ([9](#_ENREF_9)) |
|  | qnrC-R | TCCACTTTACGAGGTTCT |  |
|  | qnrD-F | TTACGGGGAATAGAGTTA | ([10](#_ENREF_10)) |
|  | qnrD-R | AATCAGCCAAAGACCAAT |  |
|  | qnrS-F | CAATCATACATATAGGCACC | ([8](#_ENREF_8)) |
|  | qnrS-R | TCAGGATAAACAACAATACCC |  |
|  | qepA-F | CCAGCTCGGCAACTTGATAC | ([11](#_ENREF_11)) |
|  | qepA-R | ATGCTCGCCTTCCAGAAAA |  |
|  | aac6'-F | TTGCGATGCTCTATGAGTGGCTA | ([12](#_ENREF_12)) |
|  | aac6'-R | CTCGAATGCCTGGCGTGTTT |  |
| cmeABC | CmeABC-F | CCAGAAGCTGTAGCTTTTGGT | This study |
|  | CmeABC-R | GCCTGCTATTTACAAGGCT |  |
| DH161cmeABC | cmeAKpnI-F | AAGGTACCTGTTGAATTTGTTGTAAATG | This study |
|  | cmeCKpnI-R | GGGCGGTACCTTATTCTTATTTTGATAAG |  |

References

1. **Ma L, Shen Z, Naren G, Li H, Xia X, Wu C, Shen J, Zhang Q, Wang Y.** 2014. Identification of a novel G2073A mutation in 23S rRNA in amphenicol-selected mutants of *Campylobacter jejuni*. PLoS One **9:**e94503.

2. **Gibreel A, Kos VN, Keelan M, Trieber CA, Levesque S, Michaud S, Taylor DE.** 2005. Macrolide resistance in *Campylobacter jejuni* and *Campylobacter coli*: molecular mechanism and stability of the resistance phenotype. Antimicrob Agents Chemother **49:**2753-2759.

3. **Gordon L, Cloeckaert A, Doublet B, Schwarz S, Bouju-Albert A, Ganiere JP, Le Bris H, Le Fleche-Mateos A, Giraud E.** 2008. Complete sequence of the *floR*-carrying multiresistance plasmid pAB5S9 from freshwater *Aeromonas bestiarum*. J Antimicrob Chemother **62:**65-71.

4. **Kehrenberg C, Schwarz S.** 2006. Distribution of florfenicol resistance genes *fexA* and cfr among chloramphenicol-resistant *Staphylococcus* isolates. Antimicrob Agents Chemother **50:**1156-1163.

5. **Wang Y, Lv Y, Cai J, Schwarz S, Cui L, Hu Z, Zhang R, Li J, Zhao Q, He T, Wang D, Wang Z, Shen Y, Li Y, Fessler AT, Wu C, Yu H, Deng X, Xia X, Shen J.** 2015. A novel gene, *optrA,* that confers transferable resistance to oxazolidinones and phenicols and its presence in *Enterococcus faecalis* and *Enterococcus faecium* of human and animal origin. J Antimicrob Chemother **70:**2182-2190.

6. **Zirnstein G, Helsel L, Li Y, Swaminathan B, Besser J.** 2000. Characterization of *gyrA* mutations associated with fluoroquinolone resistance in *Campylobacter coli* by DNA sequence analysis and MAMA PCR. FEMS Microbiol Lett **190:**1-7.

7. **Gibreel A, Sjogren E, Kaijser B, Wretlind B, Skold O.** 1998. Rapid emergence of high-level resistance to quinolones in *Campylobacter jejuni* associated with mutational changes in gyrA and parC. Antimicrob Agents Chemother **42:**3276-3278.

8. **Cattoir V, Poirel L, Rotimi V, Soussy CJ, Nordmann P.** 2007. Multiplex PCR for detection of plasmid-mediated quinolone resistance qnr genes in ESBL-producing enterobacterial isolates. J Antimicrob Chemother **60:**394-397.

9. **Kim HB, Park CH, Kim CJ, Kim EC, Jacoby GA, Hooper DC.** 2009. Prevalence of plasmid-mediated quinolone resistance determinants over a 9-year period. Antimicrob Agents Chemother **53:**639-645.

10. **Adachi F, Yamamoto A, Takakura K, Kawahara R.** 2013. Occurrence of fluoroquinolones and fluoroquinolone-resistance genes in the aquatic environment. Sci Total Environ **444:**508-514.

11. **Cano ME, Rodriguez-Martinez JM, Aguero J, Pascual A, Calvo J, Garcia-Lobo JM, Velasco C, Francia MV, Martinez-Martinez L.** 2009. Detection of plasmid-mediated quinolone resistance genes in clinical isolates of Enterobacter spp. in Spain. J Clin Microbiol **47:**2033-2039.

12. **Park CH, Robicsek A, Jacoby GA, Sahm D, Hooper DC.** 2006. Prevalence in the United States of *aac(6')-Ib-cr* encoding a ciprofloxacin-modifying enzyme. Antimicrob Agents Chemother **50:**3953-3955.

13. **Lin J, Cagliero C, Guo B, Barton YW, Maurel MC, Payot S, Zhang Q.** 2005. Bile salts modulate expression of the CmeABC multidrug efflux pump in *Campylobacter jejuni*. J Bacteriol **187:**7417-7424.
